# Structures of soluble guanylate cyclase: implications for regulatory mechanisms and drug development

**DOI:** 10.1042/BST20130228

**Published:** 2014-01-23

**Authors:** Opher Gileadi

**Affiliations:** *Structural Genomics Consortium, University of Oxford, Old Road Campus Research Building, Roosevelt Drive, Oxford OX3 7DQ, U.K.

**Keywords:** allosteric regulation, cGMP, guanylate cyclase, hypertension, nitric oxide, vasodilation, AC, adenylate cyclase, ANF, atrial natriuretic factor, BNF, brain natriuretic factor, CC, coiled coil, GC, guanylate cyclase, HNOX, haem-containing NO/oxygen-binding, PAS, Per/Arnt/Sim, pGC, membrane-spanning (particulate) GCs, sGC, soluble GC

## Abstract

Activation of cGMP synthesis leads to vasodilation, and is an important mechanism in clinical treatment of angina, heart failure, and severe peripheral and pulmonary hypertension. The nitric oxide-responsive sGC (soluble guanylate cyclase) has been the target of recent drug discovery efforts. The present review surveys recent data on the structure and regulation of sGC, and the prospects of new avenues for therapeutic intervention.

## Introduction

Nucleotide cyclases generate two of the most important intracellular messengers, cAMP and cGMP. cGMP causes vasodilation by relaxing vascular smooth muscle cells. It is also involved in reducing platelet aggregation, in neuronal transmission, in vision and in cancer [1–3]. cGMP may also have a more general role in regulating energy expenditure in cells [4].

cGMP in animal cells is generated by two classes of GCs (guanylate cyclases): sGC (soluble GC) and pGC [membrane-spanning (particulate) GC]. sGC is the receptor for nitric oxide (NO). pGCs are transmembrane receptors activated by the peptide natriuretic factors ANF (atrial natriuretic factor) and BNF (brain natriuretic factor). NO acts in a paracrine manner, secreted by endothelial cells in response to hypoxia and/or mechanical stimuli, and activating sGC in adjacent smooth muscle cells. ANF and BNF are secreted from the heart atrium and brain respectively, and act systemically on ANF receptors. Upon hormone binding, the GC of the intracellular domains is activated, leading to increased cGMP levels. cGMP then activates downstream effectors (cGMP-dependent protein kinase, Ca^2+^ channels and phosphodiesterases) to eventually cause muscle relaxation and vasodilation.

sGC is the target of the earliest class of cardiovascular drugs known, nitroglycerin and other NO precursors, which have been used since the 19th Century as vasodilators to alleviate angina, myocardial infarction and other disorders [2,5]. In recent years, additional drugs with more favourable pharmacology have entered into clinical trials [6]. The structure, regulation and pharmacology of sGC are the subject of a vast literature (see, for example, [7]). In the present review, we survey recent advances in the structural biology of sGC and their possible implications on mechanistic understanding and opportunities for developing new types of modulators.

## Structural organization of GCs

**Figure 1:**
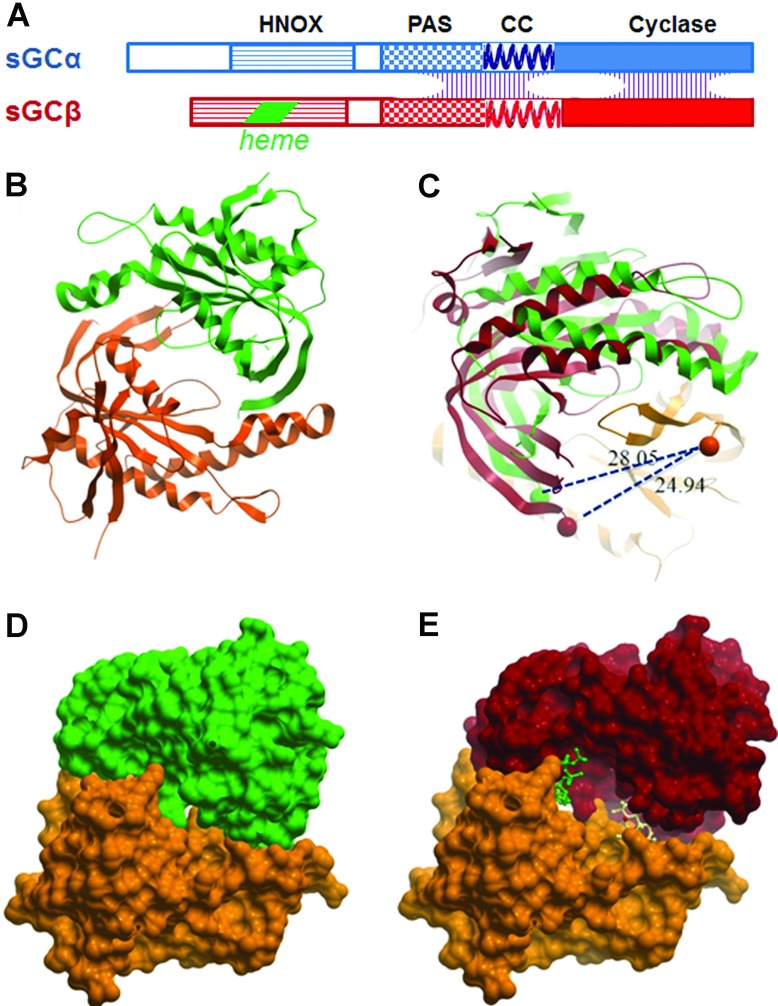
The catalytic domain of human sGC (**A**) Domain organization of the subunits of sGC. Dimerization interfaces are spread along the catalytic, CC and PAS domains. (**B**) Ribbon diagram of the catalytic domains in the crystal structure of human sGC (PDB code 3UVJ) (α is green, and β is orange). (**C**) Viewed from above, a rotation of the α subunit between the inactive conformation (green) and an active conformation (red) modelled after the structure of AC. The spheres present the first amino acids of the catalytic domains, showing that the conformational shift results in a change in the distance between the α and β N-termini from 28 to 25 Å, which should affect the interaction with the CC. (**D**) Space-filling model of the inactive conformation. (**E**) Model of the active conformation, showing cavities for the substrate GTP (modelled in green) and a potential allosteric regulator at the pseudosymmetric site (depicted in white). These cavities are collapsed in the inactive crystal structure (**D**).

GCs of animal cells, as well as ACs (adenylate cyclases), derive from ancestral enzymes consisting of homodimers of catalytic domains; they have evolved into fusion proteins with regulatory or membrane-anchoring domains [8]. ACs include two catalytic half-domains within the same polypeptide, whereas GCs are either homodimers (e.g. GC-A and GC-B, the natriuretic factor receptors) or heterodimers (sGC). Human sGC is composed of two homologous subunits: α1 and β1. The domain organization of sGC is depicted schematically in Figure 1(A). The C-terminal domains of both subunits combine to form a heterodimeric catalytic domain. In addition, both sGC subunits include three distinct regulatory and structural domains: an N-terminal HNOX (haem-containing NO/oxygen-binding) domain, a PAS (Per/Arnt/Sim)-like domain, and an α-helical region capable of forming CCs (coiled coils). Only the β subunit binds haem; the corresponding region of the α subunit may have a similar structure, but lacks critical haem-binding residues. The dimer interface is complex, and involves the catalytic, CC and PAS domains. Heterodimers seem to be the preferred form in cells, but the features that determine this preference, and any possible role for homodimers, are not fully defined.

## Structures of sGC domains

The catalytic core of sGC is composed of two homologous domains. The active site lies at the interface between the two domains. Ligand binding and catalysis require the precise alignment of residues from both subunits; this is very sensitive to relative motion of the subunits as well as to local changes, which underlie the modes of allosteric regulation. We have reported recently a crystal structure of the catalytic domain of human sGC [9]. Structures of GCs of algae and cyanobacteria have been reported previously [10,11], in addition to multiple structures of mammalian and microbial ACs.

The studies of the catalytic domains have revealed the following features.

(i) The isolated catalytic domains of both α and β subunits can form stable homodimers, both of which are enzymatically inactive. When mixed at high concentrations, the subunits can exchange to form heterodimers, which are active even in the absence of NO. Crystal structures were obtained for both the α–β heterodimer and homodimers of the β catalytic domain [9]. The dimer interfaces are similar between the heterodimers and the β homodimers. This promiscuity of subunit association is a feature of cyclase catalytic domains; for example, active mammalian ACs have been assembled by combining catalytic domains from AC 1 or 5 with AC 2 [12,13].
(ii) The two subunits are arranged as a wreath-like head-to-tail dimer (Figure 1B). This arrangement has an inherent pseudosymmetry, which could create a second pseudosymmetric active site (not shown). In sGC, the pseudosymmetric site lacks the critical residues for catalysis and is therefore not enzymatically active.
(iii) The crystal structure of the heterodimer is in an inactive conformation: the active-site residues from the two subunits are misaligned, and the cavity that should contain the substrate is not formed.
(iv) It is possible to model an active conformation by aligning the α and β subunits separately with the two subdomains of an active AC structure. This entails a 26° rigid-body rotation of the α subunit (Figure 1C). In this conformation, it is possible to model a molecule of GTP bound at the substrate site, with most of the substrate-binding and catalytic residues in the same positions as in AC (see Figure 4 of [9]). Space-filling models of both conformations (Figures 1D and 1E) show that the conformational change generates a cavity that can contain a GTP molecule (modelled in green), as well as a pseudosymmetry-related cavity, which can engulf a small molecule (illustrated by a white atomic model). Structures of AC with the allosteric activator forskolin [12,14] have shown precisely the same arrangement, with the active and pseudosymmetry sites occupied by ATP analogues and forskolin respectively. This immediately suggests a mechanism for direct allosteric regulation of the catalytic domain through binding of small molecules to the pseudosymmetry site, stabilizing or inducing the transition to an active conformation.
(v) The question arises whether the inactive conformation of the catalytic subunits seen in the crystal ever exists in the context of the full-length protein. The catalytic domains are linked directly to an upstream CC domain, which is expected to be quite rigid. As seen in Figure 1(C), the distance between the N-termini of the catalytic α and β domains changes from 28.05 to 24.94 Å (1 Å=0.1 nm) between the inactive and active conformations. One can imagine that the distance is determined by the arrangement of the CC region, which can favour a constitutive structure similar to that of the active conformation; more intriguingly, this could be a mechanism for transmitting regulatory influences from the upstream haem-binding domains to the catalytic domain, via changes in the intervening CC region.

Other regions of sGC have been crystallized successfully. The CC region preceding the catalytic domain of the β subunit has been crystallized as a four-helix antiparallel bundle [15]. The authors indicate that an antiparallel organization of the CC region is unlikely to occur within the full-length protein, as it places the C-termini of the two helices at a distance that is much greater than the distance between the N-termini of the catalytic domain. On the basis of the distribution of charged residues in the α-helical regions of the α and β subunits, the authors suggest a preference for an antiparallel orientation in the heterodimer. This has been corroborated by cross-linking analysis [16]. Interestingly, this suggests that linking together two domains (the catalytic and CC domains), either of which can readily form homodimers, can lead to a strong preference for heterodimerization.

The structure of a PAS domain of sGC from the moth *Manduca sexta* was published recently [17]. Although the structure does not immediately shed light on the association or regulatory roles of the PAS domain, comparison with other PAS domains suggests that a mobile region within this domain may generate a cavity large enough to bind small molecules. The same group studied the arrangement of the entire regulatory domain (HNOX–PAS–CC) by chemical cross-linking and SAXS [16]. This enabled them to build a rough model in which the HNOX and PAS domains are folded back along a backbone of the CC domain, poised for direct interaction with the catalytic domains (which were not present in the protein fragments used). It would be interesting to see how regulatory ligands such as NO, cinaciguat or riociguat (see below) affect the gross structure of sGC.

Finally, HNOX domains of sGC have not been crystallized, but structures of highly homologous domains from cyanobacteria have been solved [18–20]. These structures provide important insights on the mode of binding of NO, the discrimination between ligands, and the structural consequences of NO binding, as well as of haem-mimetic drugs [21]. Briefly, NO binding releases an iron-ligated histidine (His^105^) on the distal face of the haem group. This leads to structural changes in helix αF that contains His^105^, which are thought to be critical in transducing the signals to the catalytic domain. The important role of this region of the HNOX domain in cyclase activation is supported by mutagenesis and by hydrogen–deuterium exchange analysis [22–24].

## Regulation of GC activity

The simplest regulatory scheme for sGC envisions inherently active catalytic domains which are inhibited by the N-terminal domains in the absence of NO. NO binding to the haem causes a structural change in the HNOX domain which is allosterically transmitted to the catalytic domain, leading to relief of inhibition. Support for this scheme comes from the observation that the isolated catalytic domains of α1 and β1, when mixed, exhibited concentration-dependent cyclase activity; this activity could be inhibited *in trans* by adding large concentrations of the purified regulatory domain [25]. A protein fragment containing the HNOX, PAS and part of the CC domain (β-1–385) was considerably more effective than the isolated HNOX domain (β-1–194). However, these experiments do not fully reflect the complexities of interdomain interactions in the intact proteins. Primarily, inhibition *in trans* is not relieved by NO [25]. Furthermore, mutagenesis of the HNOX domain usually lead to loss of activation, but only one mutation (of Thr^110^) reportedly led to a modest increase in the basal (i.e. NO-independent) cyclase activity of the full-length protein [22]. Another mutation in the catalytic domain (α C541S) also led to an increase in basal activity, but this may be through a direct effect on the conformation of the catalytic region [23,26]. A thorough investigation of the interactions between the different domains of rat sGC was carried out using hydrogen–deuterium exchange kinetics. Adding the HNOX and PAS domains (β-1–384) *in trans* resulted in localized changes in hydrogen accessibility in the catalytic domains of both the α and β subunits, primarily in the ‘front’ side; this footprint is consistent with the HNOX/PAS domains blocking substrate entry to the active site. Notably, when comparing the hydrogen–deuterium exchange pattern of the isolated catalytic domains with the full-length protein (in which the N-terminal region is present *in cis*), the footprint on the β subunit is shifted, indicating a difference in the interaction interface.

The primary activation switch of sGC is the binding of NO to a sixth co-ordination site of the haem iron in the HNOX domain of the β subunit and subsequent breaking of the bond to His^105^. However, this is not the whole mechanism, as binding of a single molecule of NO leads to only a modest activation, which is enhanced severalfold by binding of additional NO molecule(s) to lower-affinity sites [20,27–29]; the location of these additional sites is not clear, and may be either at the haem iron or a protein cysteine residue. sGC can be desensitized by a number of mechanisms. Oxidation of the haem iron to Fe(III), which may be followed by the loss of the haem group, leads to inactivation and subsequent degradation. In addition, other less well-defined events such as oxidation or nitrosylation of critical cysteine residues may also lead to enzyme inactivation.

The development of drugs that allosterically activate sGC was initiated following the serendipitous discovery of YC-1, which can stimulate cyclase activity 30–40-fold in the absence of NO, but can also synergize with NO. Activation by YC-1 required the presence of Fe(II)-haem. Extensive efforts to improve the potency, selectivity and pharmacological properties led to the development of two classes of sGC modulators [6,30]. The first, which includes YC-1 and the drug candidate riociguat, are termed ‘sGC stimulators’ and require haem. A distinct class of molecules termed ‘sGC activators’, which include the drug candidate cinaciguat, are haem-independent. Crystallographic studies [21,31] showed that cinaciguat and another activator (BAY 60-2770) replace the haem in a bacterial HNOX protein, presumably generating a conformation that is comparable with that of an activated haem–NO complex. The mechanism of action of YC-1 and other stimulators is less clear, and may involve stabilization of bound NO as well as allosteric effects on the global structure of sGC.

**Figure 2:**
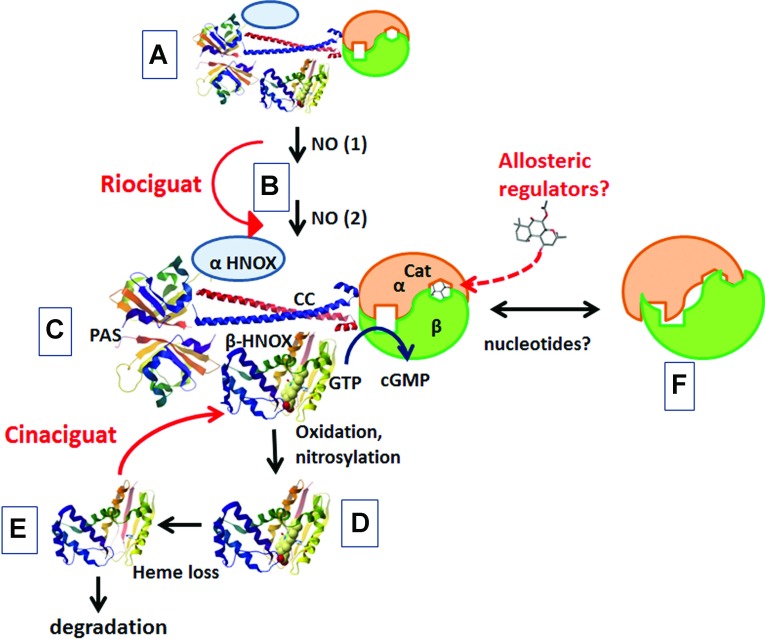
Scheme of the conformational and chemical changes in sGC See the text for details.

Figure 2 presents a schematic summary of some of the states and transitions of sGC. Figure 2(A) represents basal state, without NO. Binding of one (Figure 2B) or more (Figure 2C) NO molecules leads to full activation; this is achieved through a scission of the Fe–His^105^ bond and additional covalent and conformational changes in the HNOX domain, which are transmitted to the catalytic domains through a combination of direct contacts and other long-range interactions. The enzyme may be inactivated by oxidation, nitrosylation (Figure 2D) and haem loss (Figure 2E). Allosteric modulators can mimic the activated state either by replacing the haem moiety (cinaciguat) or by enhancing or bypassing a haem-dependent activation step (riociguat).

Figure 2(F) represents a hypothetical state of the catalytic domains, which is suggested by the crystal structure of the isolated domains. In this state, the α and β catalytic fragments are misaligned, disrupting both the catalytic site and a putative pseudosymmetric binding site. At present, it is not clear whether such a conformation exists in the full-length protein, and whether it, in fact, represents the conformation of the catalytic domain in Figure 2(A) or 2(E). It is tempting to speculate that sGC activation does include a relative movement of the two half-domains, possibly transmitted through changes in the CC domain or in the multiple interdomain interactions. If this is the case, there is a distinct possibility that small molecules that bind to the pseudosymmetric site can allosterically activate cyclase activity in a manner that is distinct from that of the current stimulators and activators.

## The case for allosteric regulation at the catalytic domain

We now turn to allosteric effects at the level of the isolated catalytic domain. In pGC, the dependence of the cyclase activity on GTP concentration shows a sigmoidal curve with a Hill coefficient of 1.5. This is taken as evidence of co-operative binding to two sites, probably the catalytic and the pseudosymmetry sites. ATP acts as an activator of cyclase activity; in the presence of ANF, ATP converts the GTP dependence into a Hill coefficient near 1, and decreases the *K*_m_. Interestingly, this implies a non-equivalence between the two nucleotide-binding sites [32]. There may be an additional regulatory role for ATP through a kinase homology domain in pGC [33,34].

It is instructive to compare the situation with ACs in which the catalytic core is composed of two subdomains within a single polypeptide. The plant diterpene forskolin is a signal-independent activator of AC, which binds in the pseudosymmetry site. It may work by stabilizing the association of the two subdomains; indeed, all crystal structures of mammalian ACs contain forskolin analogues in the pseudosymmetric site. As we do not know the structure in the absence of forskolin, it is possible that the drug not only stabilizes the complex, but also favours a subset of possible conformations of the dimer.

As for sGC, ATP and 2′-substituted analogues have been found to inhibit GC activity, at physiological concentrations of ATP [4,35]. Inhibition by ATP has been shown both in the NO-stimulated full-length enzyme and in the isolated catalytic domains [36]. This has been suggested as a mechanism for modulating the response to paracrine or autocrine NO signalling by the energy status of the cell, to regulate energy economy [4]. Analyses with multiple nucleotides and analogues [36,37] indicate a distinct regulatory nucleotide-binding site which is most likely to be located in the catalytic domain.

## Outlook: utilization of regulatory interactions for drug development

The pharmacology of cardiovascular diseases is extremely complex, reflecting the need to modulate different components of the system in a specific temporal and spatial manner, and interact with endogenous regulators which are patient-specific. There is no doubt that sGC is an attractive target for cardiovascular disorders, ranging from angina and heart failure to peripheral and pulmonary hypertension [6,38]. Even attempts to modulate this single target face issues of delivery, desensitization and side effects [39]. Hence the drive to develop new compounds that utilize different states or regulatory mechanisms; as mentioned, haem-dependent and -independent allosteric regulators (riociguat and cinaciguat) are in clinical testing, as alternatives to the traditional NO-generating drugs [40–42]. The advances in characterization of sGC suggest that new chemistry directed at allosteric sites within the catalytic domain may provide further options for modulating cGMP generation. There are vast libraries of nucleotide-mimetic compounds which could provide initial hits; the recent advances in crystallization could form the basis of fragment screening and the application of computational methods to this search.
